# 4D-flow MRI derived wall shear stress for the risk stratification of bicuspid aortic valve aortopathy: A systematic review

**DOI:** 10.3389/fcvm.2022.1075833

**Published:** 2023-01-09

**Authors:** Jiaxing Jason Qin, Peyman Obeidy, Mustafa Gok, Alireza Gholipour, Stuart M. Grieve

**Affiliations:** ^1^Imaging and Phenotyping Laboratory, Charles Perkins Centre, University of Sydney, Sydney, NSW, Australia; ^2^Sydney Medical School and School of Health Sciences, Faculty of Medicine and Health, University of Sydney, Sydney, NSW, Australia; ^3^Department of Radiology, Faculty of Medicine, Aydın Adnan Menderes University, Aydın, Turkey

**Keywords:** 4D-flow MRI, wall shear stress, bicuspid aortic valve, ascending aorta, aortic dilatation

## Abstract

**Purpose:**

Current intervention guidelines for bicuspid aortic valve (BAV) associated ascending aorta (AAo) dilatation are suboptimal predictors of clinical outcome. There is growing interest in identifying better biomarkers such as wall shear stress (WSS) to help risk stratify BAV aortopathy. The aim of the systematic review is to synthesize existing evidence of the relationship between WSS and aortopathy in the BAV population.

**Methods:**

A comprehensive literature search of available major databases was performed in May 2022 to include studies that used four-dimensional flow cardiac magnetic resonance (4D-flow) MRI to quantify WSS in the AAo in adult BAV populations. Summary results and statistical analysis were provided for key numerical results. A narrative summary was provided to assess similarities between studies.

**Results:**

A total of 26 studies that satisfied selection criteria and quality assessment were included in the review. The presence of BAV resulted in significantly elevated WSS magnitude and circumferential WSS, but not axial WSS. The presence of aortic stenosis had additional impact on WSS and flow alterations. BAV phenotypes were associated with different WSS distributions and flow profiles. Altered protein expression in the AAo wall associated with WSS supported the contribution of altered hemodynamics to aortopathy in addition to genetic factors.

**Conclusion:**

WSS has the potential to be a valid biomarker for BAV aortopathy. Future work would benefit from larger study cohorts with longitudinal evaluations to further characterize WSS association with aortopathy, mortality, and morbidities.

**Systematic review registration:**

https://www.crd.york.ac.uk/prospero/display_record.php?ID=CRD42022337077, identifier CRD42022337077.

## 1. Introduction

Bicuspid aortic valve (BAV) is the most common congenital valvular disease with a prevalence of 1–2% (1). A significant proportion of the BAV population develops ascending aorta (AAo) dilatation during their lifetime, which predisposes them to acute aortic syndromes including dissection and rupture with significant mortality risks (1, 2). According to existing clinical guidelines, prophylactic surgical intervention is indicated when aortic diameter is greater than 5.5 cm in the BAV population, or lower (>5.0 cm) if there are concomitant risk factors (family history, annual growth rate >3 mm/year, severe aortic regurgitation, connective tissue disorders) (2–4). However, evidence supporting the size-guided prediction of acute aortic syndromes and management of aortic dilatation remains lacking especially in aortic diameters less than 5.5 cm (5), and acute aortic dissection and rupture is known to also occur in people with normal sized aorta (2, 6, 7). Previously, the development of aortopathy was considered secondary to underlying genetic abnormalities resulting in a fragile aortic wall. However, increasingly the role of hemodynamic abnormalities associated with BAV is being recognized as an important factor in pathogenesis (8–11). There is growing interest in identifying flow-related measurements which may help better risk-stratify BAV patients and predict acute aortic syndromes (2).

**FIGURE 1:**
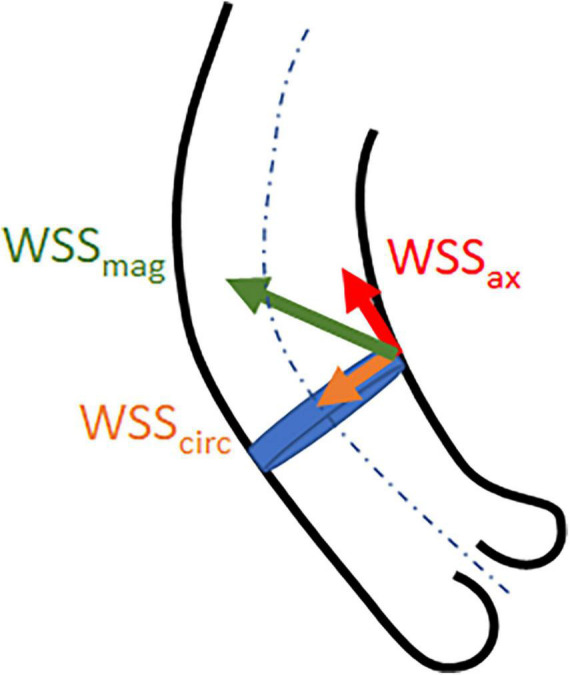
Illustrative representation of wall shear stress net magnitude (WSS_mag_) and the vector components axial (WSS_ax_) and circumferential (WSS_circ_) WSS.

The recent development in four-dimensional flow cardiac magnetic resonance (4D-flow MRI) has enabled a suite of novel flow-related parameters to be measured for the quantification of cardiac function (12). Cardiac magnetic resonance is the gold standard imaging modality in cardiovascular assessment, and is non-ionizing in contrast to Computed Tomography (13, 14). In the aorta, flow-sensitive MR with volumetric coverage allows the three-dimensional (3D) visualization of flow jet patterns, quantification of flow velocity and volume, as well as quantification of wall shear stress (WSS) (15). WSS is the tangential viscous shear force per unit exerted on the vessel wall by the adjacent moving fluid layer caused by the no-slip condition of blood flow as it approaches the inner surface of the vessel, computed as,


(1) W⁢S⁢S=μ⋅γ

where *WSS* is expressed in *Pa* or *N*/*m*^2^, μ is the blood viscosity, and γ is the blood velocity gradient adjacent to the vessel wall (16). WSS can be expressed as net magnitude wall shear stress (WSS_mag_) and its vector components axial WSS (WSS_ax_) which is the through-plane component aligned with the main flow direction, and circumferential WSS (WSS_circ_) which is the in-plane component perpendicular to the main flow direction (Figure 1) (17).

Abnormal WSS values and WSS distribution on the aortic vessel wall have been found to be associated with aortopathy and aortic valve pathologies including BAV and aortic stenosis (AS) (2). Compared to the tricuspid aortic valve (TAV) population, people with BAV have a nine-fold risk of developing dissection (1). There is a growing body of evidence to support WSS as a potential biomarker that quantifies the interaction between abnormal flow and the pathogenesis of aortopathy, thereby potentially improving the risk stratification of acute aortic syndrome in the BAV population beyond current guidelines (2, 15).

The objective of this systematic review is to synthesize existing evidence of the relationship between WSS and aortopathy in the BAV population. The review aims to identify any correlation between WSS measurements and aortic dimension in the BAV population and factors that may influence the correlation, and to identify current gaps to inform future research direction.

## 2. Materials and methods

### 2.1. Systematic review registration

This systematic review was prospectively registered (CRD42022337077) with the international database of prospectively registered systematic reviews (PROSPERO), which is an international database of prospectively registered systematic reviews in health, where there is a health-related outcome.

### 2.2. Search strategy

A systematic review of the literature was performed in May 2022 by JQ with searches carried out in electronic databases (Pubmed, Medline, Embase, and Google Scholar) for relevant articles using the Preferred Reporting Items for Systematic Reviews and Meta-Analysis (PRISMA) checklist (18). Key search terms included “Magnetic Resonance Imaging,” “Shear Stress,” “Cardiovascular Magnetic Resonance,” and “Ascending Aorta.” Reference lists of relevant studies were also reviewed for further articles.

### 2.3. Eligibility criteria

Included studies were original research in humans that used 4D-flow MRI to quantify WSS in the AAo in adult BAV populations to investigate the pathophysiological relationships with AAo dilatation. The decision to limit the review to adult populations was based on preliminary literature review which found few studies addressing the pediatric population, or studies on pediatric populations with complex congenital heart diseases which would not be comparable with studies on adult BAV populations but could be a separate review in their own right (19). Exclusion criteria included non-4D-flow techniques, aortic arch or descending aorta, non-BAV populations, studies that used WSS purely as a metric to compare surgical techniques, intervention outcomes or disease progression without any mechanistic evaluation of the relationship with underlying pathophysiology, phantom studies, computational fluid dynamics (CFD) studies, review articles, meta-analyses, letter to the editor, conference posters or abstracts, and purely technical feasibility studies.

### 2.4. Data extraction

Data extraction included first author, institution and year of publication. Key data points collected included population characteristics (age, sex, healthy, or disease states), key findings and conclusions. Peak-systolic WSS measurements reported in the studies were recorded to provide summary numerical analysis across the studies. Where available, sample mean and standard deviation of reported peak-systolic WSS_mag_, WSS_ax_, and WSS_circ_ were also recorded. Other parameters recorded were cohort size, sample mean and standard deviation of age and aortic diameter, BAV phenotype, presence of AS and/or AR, and regions of AAo where the measurements were taken such as proximal, mid and distal AAo, or anterior and posterior aspects, and greater and lesser curvatures of the aortic wall. Measurements taken at different regions of the aorta for the same cohort were treated as distinct data points in the analysis. Same measurements reported more than once in different subgroupings were considered duplicated data points and only counted once in the analysis to avoid skewing the power toward any particular study.

### 2.5. Quality assessment

Quality assessment was performed by JQ using the Critical Appraisal Skills Programme (CASP) tool for cohort studies (20). The assessment checklist consisted of 12 questions critically appraising the methodological quality of the studies to determine the extent to which the study identified and addressed the possibility of bias in its study design. A study was deemed methodologically robust therefore included in the synthesis of results if it satisfied all questions on the checklist.

### 2.6. Statistical analysis

Numerical data from the included studies were summarized using descriptive statistics. The Shapiro–Wilk test was used for testing normality with *p* > 0.05 denoting that the sample was normally distributed. Variables with non-normal distribution were summarized using median with range. Between-group comparisons were made using Wilcoxon rank sum and Kruskal–Wallis tests for variables with non-normal distribution. Sample size-weighted linear regression was used to evaluate the correlation between reported mean WSS and aortic diameter from the included studies, results were reported as regression slope (β), coefficient of determination (*R*^2^), and *p*-value. Statistical significance was accepted at *p* < 0.05. A meta-analysis was deemed inappropriate for this review as the included studies did not consistently report on the correlations between WSS and aortic size, and they were small-scale mechanistic exploratory studies without evaluation of interventional effects or diagnostic accuracy. A narrative review was provided instead to assess similarities between the studies.

## 3. Results

### 3.1. Search strategy

**FIGURE 2:**
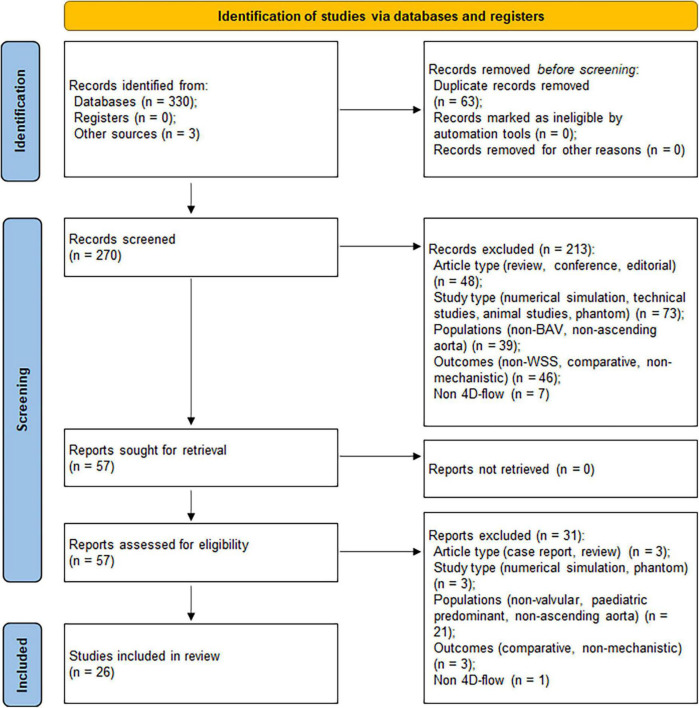
Preferred Reporting Items for Systematic Reviews and Meta-Analysis (PRISMA) flow diagram showing the article selection process (18).

An initial search in databases yielded 330 records, with further three records identified through review of references. After removal of duplicates, 270 records remained. Based on eligibility criteria, screening of the records on title and abstract resulted in 57 articles for retrieval. Further screening was conducted by reviewing the full-text which excluded a further 31 articles. A final 26 articles were included in the review based on the selection process (Figure 2).

### 3.2. Description of the included studies

All included studies were cohort studies and deemed methodologically robust based on the quality assessment using the CASP tool. A list of included studies, key WSS objectives and study cohort demographics is provided in Supplementary Table 1. There were five studies investigating the impact of functional valvular impairment (AS and/or AR) on aortic WSS (21–25); four longitudinal studies investigated the correlation of WSS and aortic growth rates (26–29). another four studies were on the topic of WSS association with aortic dilatation (10, 30–32); another four studies were on BAV phenotypes (9, 33–35); three studies investigated histopathological changes in the aortic wall associated with regions of abnormal WSS (36–40); two studies were on the association of flow patterns and WSS (39, 40); two studies investigated WSS abnormalities in BAV with normal functioning valve (41, 42). One study each on age impact of WSS (43) and using population-averaged heatmap to detect WSS abnormalities (44). 4D-flow acquisitions were performed on 1.5 T or 3 T machines. Echo time (TE) ranged between 1.5 and 4.2 ms, repetition time (TR) between 2.3 and 42 ms, and flip angle between 7 and 20°. Spatial resolution ranged between 1.6 and 4.0 mm (mean 2.7±0.7 mm) in the lowest resolution dimension, temporal resolution ranged between 27 and 77 ms (mean 44.5 ±12.8 ms).

### 3.3. Overall BAV and TAV cohort characteristics

Table 1 presents the key numerical data of the studies summarizing all distinct measurements comparing TAV and BAV cohorts. The TAV cohort included both healthy and TAV subjects with AAo dilatation. The median study sample size was 23 participants for TAV and 30 participants for BAV cohort. Across studies, reported sample mean age was similar between TAV and BAV. Reported sample mean aortic diameter was significantly larger in BAV than TAV. BAV had higher reported sample mean WSS_mag_ and WSS_circ_, and lower WSS_ax_ than TAV, however, the difference in sample mean WSS_ax_ between BAV and TAV was not statistically significant. Across studies, sample mean WSS_circ_ was consistently reported higher in BAV than TAV regardless of the presence of aortic dilatation, BAV phenotypes or the aortic region where the measurements were taken (25, 27, 31, 40, 41).

**TABLE 1:** Summary of key numerical data of the included studies.

	TAV		BAV		
	Median	Range	Median	Range	*p*
Cohort sample size	23	9–245	30	5–280	0.34
Mean age (years)	47	23–70	47	20–61	0.86
Mean aortic diameter (cm)	3.50	2.39–4.40	4.03	2.96–5.04	**<0**.**001**
Mean WSS_mag_ (N/m^2^)	0.52	0.18–1.53	0.73	0.12–2.67	**<0**.**001**
Mean WSS_ax_ (N/m^2^)	0.37	0.22–0.93	0.27	0.13–0.72	0.080
Mean WSS_cir_ (N/m^2^)	0.10	0.01–0.29	0.28	0.13–0.64	**0**.**0028**

Reported mean values from the studies were summarized using median with range. WSS_ax_, axial wall shear stress; WSS_cir_, circumferential wall shear stress; WSS_mag_, WSS magnitude. Bold values denote statistical significance.

In contrast, comparative differences of WSS_mag_ between BAV and TAV were more variable across studies where most studies found higher WSS_mag_ in BAV than TAV cohorts that appeared to be regionally dependent. Barker et al. (9) reported significantly elevated WSS_mag_ at the right-anterior aspect of mid AAo in the BAV cohort compared to healthy age-matched and age/aorta size matched TAV cohorts regardless of the presence of AS, but more comparable in other regions of the aorta. Similarly, Meierhofer et al. (41) also found higher mid and comparable distal AAo WSS_mag_ in BAV compared to TAV cohort. Six other studies (22, 25, 28, 30, 34, 39) also found higher WSS_mag_ in BAV compared to TAV cohorts especially in the proximal or right anterior aspects of the AAo. Three studies (21, 27, 40) found no difference in WSS_mag_ between BAV and TAV cohorts. These studies all had larger aorta in BAV than TAV cohort, and less regionally specific WSS_mag_ quantifications (e.g., WSS_mag_ averaged over the entire AAo). Geeraert et al. (31) reported WSS_mag_ being significantly lower in the BAV cohort compared to healthy TAV cohort when measured at the level of sinuses of Valsalva (*p* < 0.01), but comparable when measured at mid and distal AAo (*p* = 0.2 for both). The BAV cohort had significantly larger aorta than TAV at all levels of measurement but all measured mean indexed aortic diameters were less than 2.0 cm/mm^2^. The authors acknowledged the discrepancy compared to other studies and suggested image spatial resolution and segmentation quality may be the contributing factors.

### 3.4. WSS correlation with aortic diameter

**FIGURE 3:**
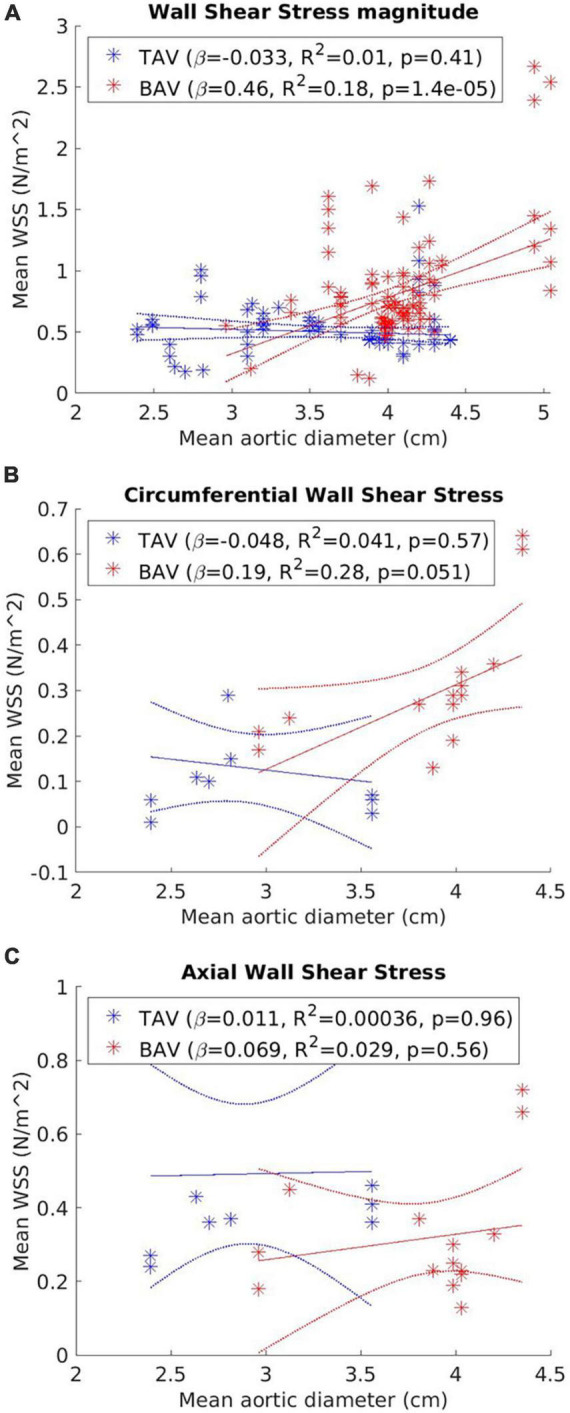
Correlation between **(A)** wall shear stress net magnitude (WSS_mag_); **(B)** circumferential WSS (WSS_circ_); and **(C)** vector components axial (WSS_ax_) and aortic diameter for tricuspid aortic valve (TAV) and bicuspid aortic valve (BAV) cohorts. Each data point represents a distinct WSS measurement. Line of best fit (solid line) and 95% confidence bounds (dotted line) from the weighted linear regression analysis are also represented.

Figure 3 presents the correlation between WSS and aortic diameter for BAV and TAV cohorts. Statistically significant moderate and positive correlations with aortic diameter were found for WSS_mag_ (*p* < 0.001) in the BAV cohort. No significant correlation was found between WSS_circ_ (*p* = 0.051) or WSS_ax_ (*p* = 0.56) and aortic diameter for the BAV cohort, or for any of the WSS metrics in the TAV cohort. However, a positive trend was observed for all WSS metrics in the BAV cohort while no trend was observed for any of the WSS metrics in the TAV cohort. The summary findings for WSS_circ_ and WSS_ax_ in the BAV cohort were consistent with individual study findings. WSS_circ_ was consistently found to be elevated in BAV compared to TAV cohorts (10, 25, 27, 40, 41), and correlated positively with aortic dilatation in BAV cohorts (26). WSS_ax_ tended to be lower in BAV than TAV cohorts (27, 40, 41), but not significantly associated with aortic dilatation (26). However, the summary findings for WSS_mag_ in the BAV cohort appeared to be in discordance with findings reported in individual studies. Three studies found no statistically significant difference in WSS_mag_ between BAV patients with vs. without aortic dilatation, however, WSS_mag_ tended to be lower in BAV with aortic dilatation (30, 32, 39). Four studies found significantly lower WSS_mag_ in larger aortic diameters amongst the BAV cohort (21, 28, 43, 31). One study (38) accounted for all the WSS_mag_ measurements taken at aortic diameter above two standard deviations from the mean across all the studies (i.e., >4.8 cm, Figure 3A). In this study, WSS_mag_ was measured at four regions of the proximal aortic wall and the highest value was measured along the greater curvature (2.67 N/m^2^), while the median WSS_mag_ across all the studies was 0.73 N/m^2^ (Table 1). Excluding this study from the weighted linear regression analysis resulted in no statistically significant correlation between WSS_mag_ and aortic diameter for the BAV cohort (β = 0.18, *R*^2^ = 0.029, *p* = 0.11).

**FIGURE 4:**
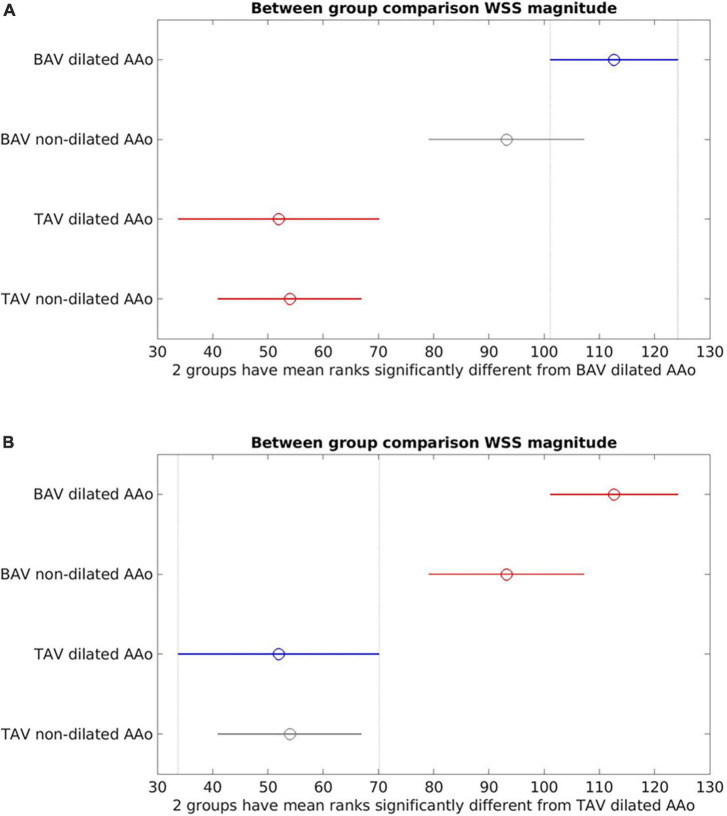
Between-group comparisons of bicuspid aortic valve (BAV) dilated ascending aorta (AAo), BAV non-dilated AAo, tricuspid aortic valve (TAV) dilated AAo and TAV non-dilated AAo. **(A)** Bicuspid aortic valve (BAV) dilated is the reference cohort (blue). No statistically significant difference between BAV dilated and BAV non-dilated (gray). There were statistically significant differences between BAV dilated and the TAV cohorts (red); **(B)** TAV dilated is the reference cohort (blue). No statistically significant differences between TAV dilated and TAV non-dilated (gray). Statistically significant difference was present between TAV dilated and the BAV cohorts (red).

Between-group comparisons (Figure 4) of dilated (aortic diameter ≥4 cm) vs. non-dilated cohorts in BAV and TAV found no statistically significant differences in WSS_mag_ between dilated and non-dilated cohorts in either BAV or TAV populations (BAV: *p* = 0.21; TAV: *p* = 0.99). Within the dilated or non-dilated cohorts, WSS_mag_ was significantly higher in BAV than TAV populations (dilated: *p* < 0.001; non-dilated: *p* = 0.0012).

### 3.5. WSS association with aortic growth

Longitudinal studies have observed an association between aortic growth rate and WSS (26), WSS angle (27) or aortic surface area with elevated WSS (29). WSS_circ_ was found to be predictive of aortic diameter growth rate (*p* = 0.014, β = 0.895) in the mid AAo in both cohort-wise and regional-wise analyses, whilst no association was found between aortic diameter growth rate and blood pressure, sex, or BAV phenotype. WSS_mag_ showed limited and sparse association (*p* = 0.046, β = 0.493) while WSS_ax_ did not show statistically significant association (*p* = 0.557) (26). Minderhoud et al. (27) found statistically significant association between volumetric growth of the entire AAo and WSS angle (angle between WSS_mag_ and WSS_ax_), WSS_circ_ and WSS_mag_ (*p* = 0.011 for each of the three metrics), adjusted for baseline volume and diastolic blood pressure, while WSS angle was the only variable significantly associated with volumetric growth of the proximal AAo (*p* = 0.031) adjusted for baseline volume and diastolic blood pressure. The authors suggested that volumetric growth rate was a better metric than the one-dimensional aortic diameter measurements due to the heterogeneous nature of AAo dilatation, and WSS angle was better at measuring the relative contribution of WSS components to the magnitude and less influenced by factors such as age and aortic size. Soulat et al. (29) observed that higher percentage of AAo area with elevated WSS_mag_ relative to healthy age- and sex-matched population average was associated with higher aortic diameter growth rate (*p* = 0.019). Maximum and mean WSS_mag_ were not predictive of faster aortic growth rate. Furthermore, there was no statistically significant association between higher aortic growth rate and age, baseline AAo diameter and aortic valve regurgitant fraction. WSS_circ_ and WSS_ax_ were not analyzed. The representation of elevated WSS on an age- and sex-adjusted heatmap was thought to be meritorious as it accounted for the influence of age on WSS values.

### 3.6. Impact of AS on WSS

**FIGURE 5:**
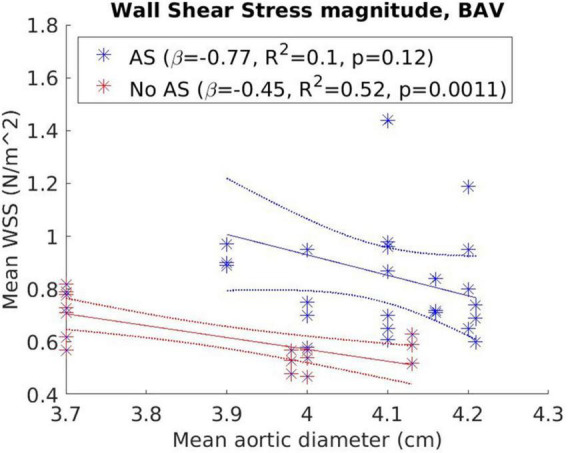
Correlation between wall shear stress magnitude (WSS_mag_) and aortic diameter in bicuspid aortic valve (BAV) cohorts with or without aortic stenosis with results from weighted linear regression also presented.

**FIGURE 6:**
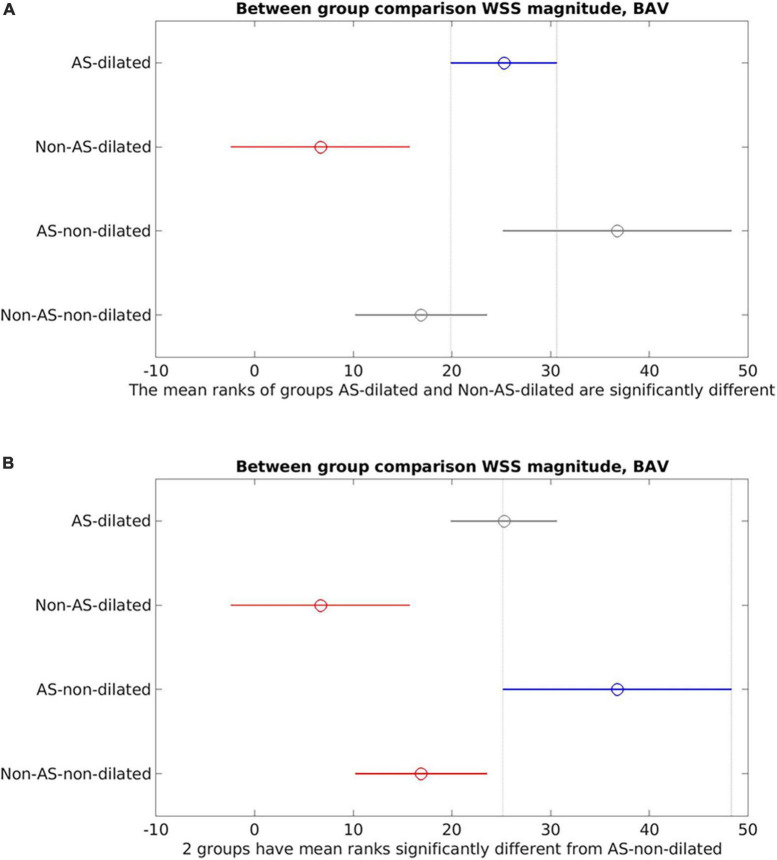
Between-group comparisons of aortic stenosis (AS)-dilated, AS-non-dilated, Non-AS-dilated, Non-AS-non-dilated in the bicuspid aortic valve (BAV) cohorts. **(A)** AS-dilated (blue) is the reference cohort. Non-AS-dilated (red) was statistically significantly different from the AS-dilated cohort. No statistically significant difference was found between AS-dilated and the non-dilated cohorts (gray). **(B)** AS-non-dilated (blue) is the reference cohort. Statistically significant difference was found between AS-non-dilated and the non-AS cohorts (red). No statistically significant difference between AS-non-dilated and AS-dilated (gray).

Figure 5 presents the correlation between WSS_mag_ and aortic diameter in BAV cohorts with or without AS. Negative trends were observed for both AS and no-AS cohorts, and the trend was statistically significant in the no-AS cohort (*p* = 0.0011; *p* = 0.12 for the AS cohort). However, available measurements adequately distributed across the aortic size spectrum was limited in the no-AS cohort hence the results should be interpreted with caution. Between-group comparisons (Figure 6) of dilated and non-dilated aorta cohorts in the presence or absence of AS in the BAV population found that the presence of AS was associated with higher WSS_mag_ (*p* = 0.006 in the dilated cohort, *p* = 0.03 in the non-dilated cohort). In the presence of AS, dilated cohort had lower WSS_mag_ than non-dilated cohort but did not reach statistical significance (*p* = 0.31). In the absence of AS, the difference between dilated and non-dilated cohorts was not significant (*p* = 0.36). In a large cohort of 515 participants, van Ooij et al. (23) investigated the impact of AS on regional WSS in TAV with aortic dilatation (TAV-TAA) and BAV cohorts. The presence of AS not only was associated with increased WSS_mag_ for all patient cohorts compared to healthy controls, it was also associated with increased variability of WSS_mag_ distribution on the aortic wall between patients. The magnitude and variability increased with increasing AS severity. In addition, in no or mild AS cohorts, distribution of elevated WSS on the aortic wall was different amongst TAV-TAA, BAV-RN (fusion of the right and non-coronary cusps) and BAV-RL (fusion of the right and left-coronary cusps) phenotypes, whilst in the moderate to severe AS cohort, WSS was similarly elevated in all aortic wall regions amongst the three patient groups. Similarly, Shan et al. (22) found significantly elevated WSS_mag_ and skewed WSS distribution in BAV with AS compared to BAV without AS. WSS_mag_ was higher along the right-anterior surface of the AAo in the BAV-AS cohort compared to the BAV-No-AS cohort. Differences were less pronounced in the left-posterior surface. There was no difference in WSS_mag_ between BAV-AS and BAV-No-AS in the proximal descending aorta. The aortic flow pattern in the BAV cohort was an eccentric helical jet extending from the aortic root to the arch resulting in uneven impingement zones on the aortic wall, compared to a central, cohesive flow pattern observed in the healthy TAV cohort. BAV-AS was also associated with highly eccentric velocity distribution which correlated closely with the WSS eccentricity (*r* = 0.9225, *p* < 0.0001). Farag et al. (21) found that AS resulted in significantly larger AAo surface area with elevated WSS than no AS in BAV patients (15 ± 11% vs. 6 ± 8%, *p* = 0.005), predominantly in the outer curvature of the AAo. Multivariate analysis showed surface area with elevated WSS increased in the presence of AS and decreased with increasing aortic diameter. The presence of AS also increased the prevalence of abnormal helical and vortical flow with increased flow eccentricity and WSS and the impact was accentuated in the presence of BAV (24).

### 3.7. WSS association with flow pattern

**FIGURE 7:**
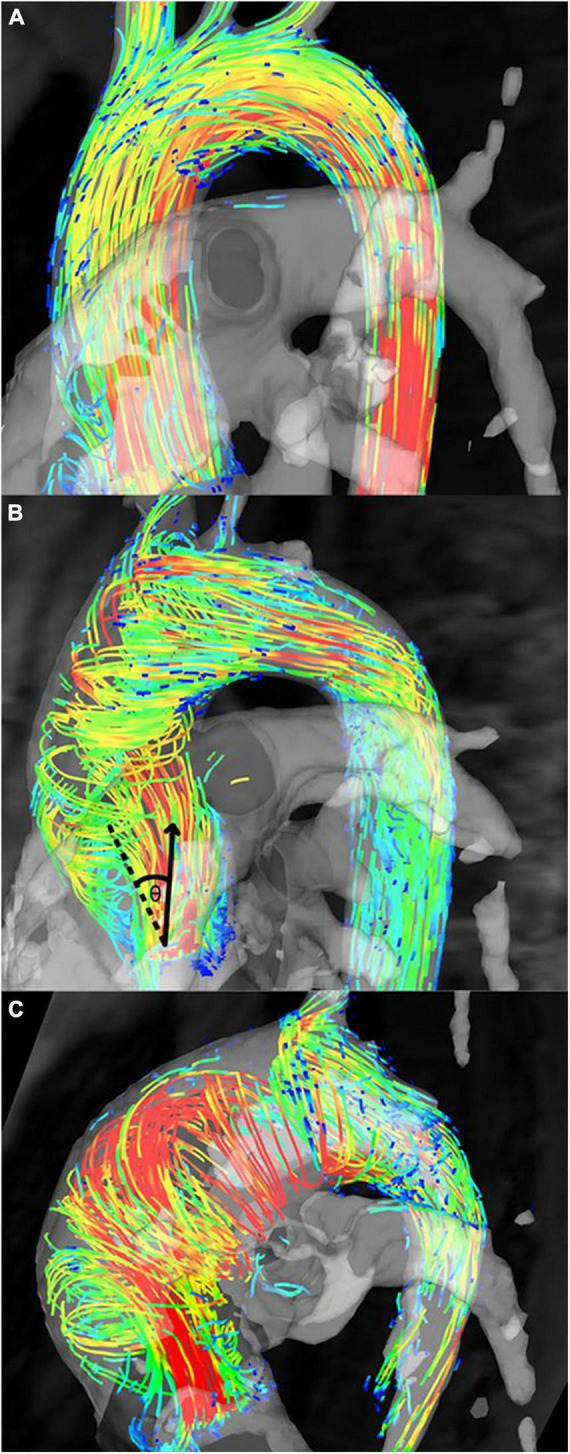
Flow patterns in bicuspid aortic valve (BAV) patients. **(A)** Normal flow pattern in subjects associated with similar aortic diameters to healthy volunteers but mildly increased rotational flow, **(B)** right-handed helical flow in subjects associated with larger aortic diameters and rotational flow values than healthy volunteers, and **(C)** left-handed helical flow in subjects associated with even larger aortic diameters than subjects in panel **(B)** and healthy volunteers (10). Permission to reproduce obtained from Wolters Kluwer Health.

Aortic flow jet pattern was found to have an association with WSS measurements and locations of elevated WSS on the aortic wall (9, 10, 22, 31, 34, 39–42, 44). When comparing BAV with different flow patterns to healthy TAV cohorts, helical flow pattern seen in BAV was associated with larger AAo diameter and significantly higher WSS_mag_ and WSS_circ_, while normal flow pattern in BAV was associated with similar AAo diameter and WSS_circ_ but marginally elevated WSS_mag_ and WSS_ax_ (Figure 7) (10, 39). Elevated WSS_circ_ in the helical flow cohorts was found to be secondary to increased rotational flow and flow eccentricity (10, 31, 41), while the predominantly laminar flow in normal flow pattern could explain the axial dominant WSS (41). Eccentric, high velocity flow impinging on the greater curvature of the AAo and recirculating flow at the lesser curvature corresponded with areas of increased and reduced WSS, respectively, compared to healthy population averaged WSS. Regions of increased WSS also corresponded with dilated AAo regions that were surgically resected (44). BAV phenotype also influenced the flow jet direction and WSS distribution. BAV-RL phenotype was found to be predominantly associated with right to right-anterior flow jets with associated elevated WSS in those regions of the proximal and mid AAo, while BAV-RN tended to result in posteriorly oriented flow jets and elevated WSS in the proximal AAo which shifted to be more anterior in the mid and distal AAo (9, 22, 33–35, 40, 42). The presence of AS did not affect flow jet direction and WSS distribution in BAV-RL (9, 34). However, in BAV-RN patients, the presence of AS resulted in peak WSS being located at the left-anterior region of the AAo while without AS, peak WSS was located at right-posterior region of the proximal AAo, although the findings were based on very small sample sizes (one to six patients) (9). In a subsequent larger study (30 patients per study cohort), the impact of AS in BAV-RN was found to be evident in the mid AAo where AS resulted in the flow jet direction and location of peak WSS to shift from right-posterior to right-anterior region of the AAo wall as flow moved from proximal to mid AAo, while no such shift was observed in BAV-RN without AS. BAV-RN also had greater impact on distal AAo with higher WSS and flow displacement than BAV-RL in this region with or without AS (34).

### 3.8. WSS and histopathology

**FIGURE 8:**
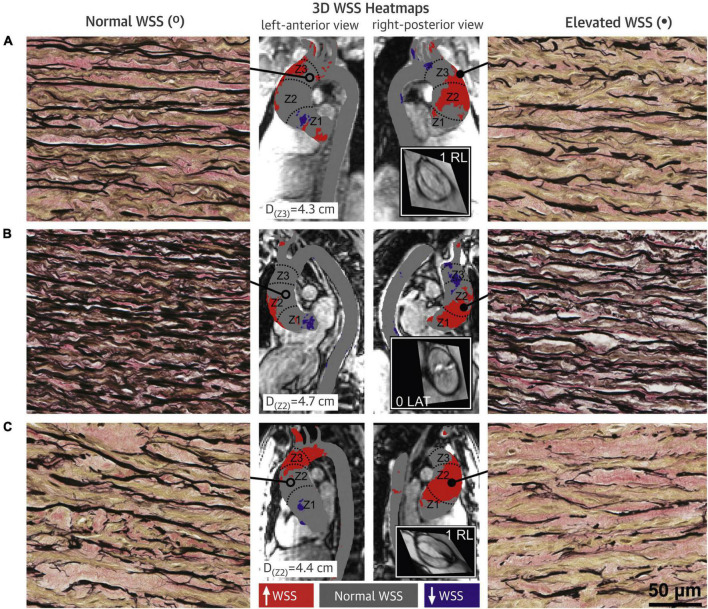
Bicuspid aortic valve (BAV) aorta wall shear stress (WSS) heatmaps and histopathology samples with elastin fiber staining. **(A–C)** Denote three different BAV aortas. Regions with elevated WSS (right panels) had fewer, thinner and more sparsely distributed elastin fibers (black) compared to regions with normal WSS (left panels). Central panels show the WSS distribution on the ascending aorta (AAo) with gray denoting normal WSS within the 95% confidence interval compared with a healthy tricuspid aortic valve population, and red and purple denoting elevated and decreased WSS, respectively (37). Permission to reproduce obtained from Elsevier.

Altered WSS was found to be associated with microscopic changes in the AAo wall (Figure 8) (36–38). A significant inverse relationship was observed between AAo wall elastic fiber thickness and regional WSS (*r* = −0.25, *p* = 0.02) (36). This relationship was also observed within each aorta, i.e., in the same individual, compared to AAo regions with normal WSS, regions with elevated WSS had decreased elastin expression with thinner and more sparsely spaced elastic fibers (37). Association between elastic fiber thickness and WSS was more prominent in the presence of AS and in smaller aortic diameters (<4.5 cm) (36). Aortopathy phenotype (root vs. ascending morphology) did not appear to have an impact on the association (38). Moreover, protein expression assessment in AAo tissue samples in BAV patients showed evidence of altered expressions of Transforming Growth Factor β (TGFβ) and Matrix Metalloproteinase (MMP). The relative concentrations of TGFβ-1 and MMP-1 were significantly higher in regions of AAo wall with elevated WSS compared to regions with normal WSS (TGFβ-1: *p* = 0.04; MMP-1: *p* = 0.03) (37). These proteins have important roles in vascular remodeling and the findings implicated the influence of altered aortic hemodynamics on molecular expression (37, 38).

## 4. Discussion

Current intervention guidelines for BAV aortopathy based on aortic size remain inadequate to account for the spectrum of the disease, and the biomechanical factors driving pathogenesis remain incompletely understood (1, 2, 11). There is ongoing effort to identify more suitable biomarkers that capture abnormal biomechanics including 4D-flow MRI derived WSS to refine the surveillance and intervention approach for aortopathy in the BAV population. In this systematic review, we summarized the existing evidence of the relationship between WSS and aortopathy in BAV populations in order to assess the potential of WSS as a biomarker for BAV aortopathy. The key findings were (1) BAV results in altered, heterogeneously distributed WSS and eccentric aberrant flow in the AAo regardless of aortic dilatation; (2) the presence of AS has additional and potentially dominant effect on altered WSS; (3) BAV phenotypes influence WSS distribution and aortic flow profile; and (4) molecular-level aortic wall remodeling is associated with altered WSS suggesting altered aortic hemodynamics at least partially contributes to the development of aortopathy in BAV.

Circumferential WSS was consistently found to be elevated in BAV compared to TAV cohorts and associated with aortic growth and helicity of aortic flow, as was reported in the individual studies. A stronger association between WSS_circ_ and aortic diameter (*R*^2^ = 0.28) than the other WSS metrics was also demonstrated in the linear regression analysis (Figure 3), although this association did not reach statistical significance (*p* = 0.051), possibly due to the small sample size. In contrast, associations between aortic dilatation and WSS_ax_ or WSS_mag_ were more inconsistent as reported by the individual studies (26, 27). WSS_ax_ was not found to be significantly associated with aortic diameter and growth and was primarily associated with central, laminar flow found in healthy populations. WSS_mag_ was generally reported as regionally averaged values, which could be impacted by the spatial heterogeneity hence affecting its reliability (45). It is also influenced by age, aortic size and the components of the WSS vector. In the summary numerical analysis (Figure 3), the exclusion of one study (32) with outlier aortic size and WSS measurements revealed no statistically significant association between WSS_mag_ and aortic diameter using data from the remaining studies. This finding did not suggest invalidity of the measurements in the excluded study, but rather suggest there may be a stepwise change in WSS in different aortic size ranges which was not revealed due to limited data. There was also insufficient data in the aortic diameters around 5 cm to evaluate the correlation between WSS measurements and surgical intervention thresholds. However, baseline AAo diameter was not found to be significantly associated with aortic growth (29), further suggesting the shortcomings of using aortic diameter as a biomarker for risk stratification (2). To better account for the heterogeneity of the WSS distribution on the aortic wall, a heatmap approach may be a better representation than using discrete or regionally averaged values (21, 23, 45). The AAo wall surface can be parameterized into standardized divisions and represented as a flat map onto which WSS measurements can be represented to enable more comprehensive evaluation and comparison of WSS values and distribution between patients (45).

The presence of AS increased WSS measurements, eccentricity and distribution, and altered flow pattern. The effect appeared to be additional to the presence of BAV and potentially becoming more dominant in more severe AS than the effects of different valvular phenotypes (23). Interestingly, by accounting for the presence of AS, a negative trend was observed between WSS_mag_ and aortic diameter (Figures 5, 6) though it did not reach statistical significance, resulting in an apparent paradox compared to when AS was not accounted for as an additional variable (Figures 3A, 4). While there was insufficient data to definitively delineate the interactions of these variables, the findings from the narrative and numerical analysis of the included studies highlighted the important role of functional valvular impairment in abnormal aortic hemodynamics, and warrants further larger scale studies to more definitively investigate the interactions between AS, aortic size and WSS components.

Bicuspid aortic valve phenotypes resulted in different WSS distribution and aortic flow profiles, and may be important in predicting regions of the aorta where faster growth and higher risks of acute aortic syndromes may occur, especially given the known asymmetrical nature of BAV aortopathy (8, 33). The varying WSS distribution associated with BAV phenotypes could potentially be better represented by the heatmap approach (45).

The increasing recognition of the contribution of altered aortic hemodynamics in addition to genetic factors in the development of aortopathy was supported by the findings of regions of altered molecular expressions associated with altered WSS within the same aorta with regions of normal WSS and protein expression. Age was known to be associated with increased vascular stiffness, increased aortic diameter and reduced WSS in healthy populations (11, 43). In addition, WSS measurements also tended to be less abnormal in the more distal parts of the aorta despite the presence of BAV or AS (9, 22, 31, 41). It may be that progressive AAo dilatation is a compensatory mechanism in an attempt to normalize local and distal WSS in the presence of natural (e.g., aging) or pathological biomechanical stimuli, and acute aortic syndromes develop when the compensation fails in the presence of persistent hemodynamic alterations originating from valvular diseases (21, 28).

There are several limitations in this systematic review. The included studies focused on BAV populations, while aortopathy and the impact of AS on aortic hemodynamics could also be important to understand in non-BAV populations. However, to include these populations in the systematic review would dilute the focus and result in a scope too broad to be feasible. Other factors associated with aortopathy such as hypertension and diabetes were not consistently accounted for across the studies and may have influenced the measurements. Quantification of WSS_mag_ showed a degree of heterogeneity amongst the included studies, in that some studies reported circumferentially averaged WSS_mag_, others reported WSS_mag_ averaged over a region of the AAo or over the entire AAo, which may have contributed to the ambiguous results. A standardized approach such as the heatmap approach would be beneficial for future inter-study comparisons. Numerical summaries and statistical analyses were provided as part of the systematic review. However, given the heterogeneity in study design and cohort characteristics, a degree of caution should be used in interpretation of the statistical results, especially where there were small or different sample sizes or unevenly distributed data points. 4D-flow MRI is known to systematically underestimate WSS due to spatial resolution limitations (39). However, this limitation was less impactful within this study as comparisons were not made with other imaging modalities, and the spatial resolutions across the included studies were sufficiently similar (within two standard deviations of mean).

The findings across the studies were suggestive of WSS having the potential to be a valid biomarker for BAV aortopathy. Future studies would benefit from larger study cohorts potentially with longitudinal evaluation to further characterize each component of WSS and their association with AAo aortopathy, mortality and morbidities, especially in the aortic diameter range around the surgical intervention threshold in order to understand how WSS can be used to risk stratify BAV aortopathy. The impact of AS would also warrant further investigations. In addition, given the limited evidence of aortic size as a reliable biomarker, it may be worthwhile to investigate any association between WSS and other cardiac function metrics such as normalized stroke volume (quotient of left ventricular stroke volume and aortic volume), which was previously shown to be associated with abnormal flow and age (11). For WSS measurements to be more widely available clinically, 4D-flow MRI also needs to be more readily performed in routine clinical workflow. Streamlined and cost effective 4D-flow MRI acquisition and analysis that is widely applicable clinically will be of the utmost importance for the characterization of WSS at a population level and the integration of WSS into clinical applications.

## Data availability statement

The original contributions presented in this study are included in this article/Supplementary material, further inquiries can be directed to the corresponding author.

## Author contributions

JQ contributed to the conceptualization, data collection and analysis, and manuscript preparation and revision. SG contributed to the conceptualization, manuscript revision, and supervision. All authors contributed to the manuscript revision, read and approved the final manuscript.

## Supplementary material

Supplementary Figure 1 Detailed database search strategy.

## References

[1] Ward C. Clinical significance of the bicuspid aortic valve. *Heart.* (2000) 83:81–5. 10.1136/heart.83.1.81 10618341 PMC1729267

[2] Adriaans B Wildberger J Westenberg J Lamb H Schalla S. Predictive imaging for thoracic aortic dissection and rupture: moving beyond diameters. *Eur Radiol.* (2019) 29:6396–404. 10.1007/s00330-019-06320-7 31278573 PMC6828629

[3] Erbel R Aboyans V Boileau C Bossone E Bartolomeo R Eggebrecht H 2014 ESC guidelines on the diagnosis and treatment of aortic diseases: document covering acute and chronic aortic diseases of the thoracic and abdominal aorta of the adult. The task force for the diagnosis and treatment of aortic diseases of the European society of cardiology (ESC). *Eur Heart J.* (2014) 35:2873–926. 10.1093/eurheartj/ehu281 25173340

[4] Tadros T Klein M Shapira O. Ascending aortic dilatation associated with bicuspid aortic valve: pathophysiology, molecular biology, and clinical implications. *Circulation.* (2009) 119:880–90. 10.1161/CIRCULATIONAHA.108.795401 19221231

[5] Otto C Nishimura R Bonow R Carabello B Erwin J Gentile F 2020 ACC/AHA guideline for the management of patients with valvular heart disease: a report of the american college of cardiology/american heart association joint committee on clinical practice guidelines. *Circulation.* (2021) 143:e72–227. 10.1161/CIR.0000000000000923 33332150

[6] Parish L Gorman J Kahn S Plappert T St John-Sutton M Bavaria J Aortic size in acute type A dissection: implications for preventive ascending aortic replacement. *Eur J Cardiothorac Surg.* (2009) 35:941–5. 10.1016/j.ejcts.2008.12.047 19237295

[7] Kim E Choi S Sung K Kim W Choe Y Oh J Aortic diameter predicts acute type A aortic dissection in patients with marfan syndrome but not in patients without marfan syndrome. *J Thorac Cardiovasc Surg.* (2014) 147:1505–10. 10.1016/j.jtcvs.2013.05.025 23879932

[8] Girdauskas E Borger M Secknus M Girdauskas G Kuntze T. Is aortopathy in bicuspid aortic valve disease a congenital defect or a result of abnormal hemodynamics? A critical reappraisal of a one-sided argument. *Eur J Cardiothorac Surg.* (2011) 39:809–14. 10.1016/j.ejcts.2011.01.001 21342769

[9] Barker A Markl M Bürk J Lorenz R Bock J Bauer S Bicuspid aortic valve is associated with altered wall shear stress in the ascending aorta. *Circ Cardiovasc Imaging.* (2012) 5:457–66. 10.1161/CIRCIMAGING.112.973370 22730420

[10] Bissell M Hess A Biasiolli L Glaze S Loudon M Pitcher A Aortic dilation in bicuspid aortic valve disease: flow pattern is a major contributor and differs with valve fusion type. *Circ Cardiovasc Imaging.* (2013) 6:499–507. 10.1161/CIRCIMAGING.113.000528 23771987 PMC3859916

[11] Callaghan F Bannon P Barin E Celemajer D Jeremy R Figtree G Age-related changes of shape and flow dynamics in healthy adult aortas: a 4D flow MRI study. *J Magn Reson Imaging.* (2019) 49:90–100. 10.1002/jmri.26210 30102443

[12] Stankovic Z Allen B Garcia J Jarvis K Markl M. 4D flow imaging with MRI. *Cardiovasc Diagn Ther.* (2014) 4:173–92. 24834414 10.3978/j.issn.2223-3652.2014.01.02 PMC3996243

[13] Hundley W Bluemke D Finn J Flamm S Fogel M Friedrich M ACCF/ACR/AHA/NASCI/SCMR 2010 expert consensus document on cardiovascular magnetic resonance: a report of the American college of cardiology foundation task force on expert consensus documents. *J Am Coll Cardiol.* (2010) 55:2614–62. 10.1016/j.jacc.2009.11.011 20513610 PMC3042771

[14] Salerno M Sharif B Arheden H Kumar A Axel L Li D Recent advances in cardiovascular magnetic resonance: techniques and applications. *Circ Cardiovasc Imaging.* (2017) 10:e003951. 10.1161/CIRCIMAGING.116.003951 28611116 PMC5777859

[15] Azarine A Garçon P Stansal A Canepa N Angelopoulos G Silvera S Four-dimensional flow MRI: principles and cardiovascular applications. *Radiographics.* (2019) 39:632–48. 10.1148/rg.2019180091 30901284

[16] Katritsis D Kaiktsis L Chaniotis A Pantos J Efstathopoulos E Marmarelis V. Wall shear stress: theoretical considerations and methods of measurement. *Prog Cardiovasc Dis.* (2007) 49:307–29. 10.1016/j.pcad.2006.11.001 17329179

[17] Sotelo J Dux-Santoy L Guala A Rodríguez-Palomares J Evangelista A Sing-Long C 3D axial and circumferential wall shear stress from 4D flow MRI data using a finite element method and a laplacian approach. *Magn Reson Med.* (2018) 79:2816–23. 10.1002/mrm.26927 28980342

[18] Page M McKenzie J Bossuyt P Boutron I Hoffmann T Mulrow C The PRISMA 2020 statement: an updated guideline for reporting systematic reviews. *BMJ.* (2021) 372:n71. 10.1136/bmj.n71 33782057 PMC8005924

[19] Allen B van Ooij P Barker A Carr M Gabbour M Schnell S Thoracic aorta 3D hemodynamics in pediatric and young adult patients with bicuspid aortic valve. *J Magn Reson Imaging.* (2015) 42:954–63. 10.1002/jmri.24847 25644073 PMC4511732

[20] Critical Appraisal Skills Programme [CASP],. *CASP cohort studies checklist.* (2022). Available online at: https://casp-uk.net/ (accessed May 20, 2022).

[21] Farag E van Ooij P Planken R Dukker K de Heer F Bouma B Aortic valve stenosis and aortic diameters determine the extent of increased wall shear stress in bicuspid aortic valve disease. *J Magn Reson Imaging.* (2018) 48:522–30. 10.1002/jmri.25956 29451963 PMC6099246

[22] Shan Y Li J Wang Y Wu B Barker A Markl M Aortic shear stress in patients with bicuspid aortic valve with stenosis and insufficiency. *J Thorac Cardiovasc Surg.* (2017) 153:1263–72.e1. 10.1016/j.jtcvs.2016.12.059 28268004 PMC5438758

[23] van Ooij P Markl M Collins J Carr J Rigsby C Bonow R Aortic valve stenosis alters expression of regional aortic wall shear stress: new insights from a 4-dimensional flow magnetic resonance imaging study of 571 subjects. *J Am Heart Assoc.* (2017) 6:e005959. 10.1161/JAHA.117.005959 28903936 PMC5634265

[24] von Knobelsdorff-Brenkenhoff F Karunaharamoorthy A Trauzeddel R Barker A Blaszczyk E Markl M Evaluation of aortic blood flow and wall shear stress in aortic stenosis and its association with left ventricular remodeling. *Circ Cardiovasc Imaging.* (2016) 9:e004038. 10.1161/CIRCIMAGING.115.004038 26917824 PMC4772425

[25] Rizk J Latus H Shehu N Mkrtchyan N Zimmermann J Martinoff S Elevated diastolic wall shear stress in regurgitant semilunar valvular lesions. *J Magn Reson Imaging.* (2019) 50:763–70. 10.1002/jmri.26680 30714251

[26] Guala A Dux-Santoy L Teixido-Tura G Ruiz-Muñoz A Galian-Gay L Servato M Wall shear stress predicts aortic dilation in patients with bicuspid aortic valve. *JACC Cardiovasc Imaging.* (2022) 15:46–56. 10.1016/j.jcmg.2021.09.023 34801463

[27] Minderhoud S Roos-Hesselink J Chelu R Bons L Van Den Hoven A Korteland S Wall shear stress angle determines aortic growth in patients with bicuspid aortic valves. *Eur Heart J Cardiovasc Imaging.* (2021) 22:1680–9. 10.1093/ehjci/jeab090.120 PMC9671285 34977931

[28] Rahman O Scott M Bollache E Suwa K Collins J Carr J Interval changes in aortic peak velocity and wall shear stress in patients with bicuspid aortic valve disease. *Int J Cardiovasc Imaging.* (2019) 35:1925–34. 10.1007/s10554-019-01632-7 31144256 PMC6995350

[29] Soulat G Scott M Allen B Avery R Bonow R Malaisrie S Association of regional wall shear stress and progressive ascending aorta dilation in bicuspid aortic valve. *JACC Cardiovasc Imaging.* (2022) 15:33–42. 10.1016/j.jcmg.2021.06.020 34419402 PMC8741630

[30] Dux-Santoy L Guala A Sotelo J Uribe S Teixidó-Turà G Ruiz-Muñoz A Low and oscillatory wall shear stress is not related to aortic dilation in patients with bicuspid aortic valve: a time-resolved 3-dimensional phase-contrast magnetic resonance imaging study. *Arterioscler Thromb Vasc Biol.* (2020) 40:e10–20. 10.1161/ATVBAHA.120.314057 31801375 PMC7771642

[31] Geeraert P Jamalidinan F Fatehi Hassanabad A Sojoudi A Bristow M Lydell C Bicuspid aortic valve disease is associated with abnormal wall shear stress, viscous energy loss, and pressure drop within the ascending thoracic aorta: a cross-sectional study. *Medicine.* (2021) 100:e26518. 10.1097/MD.0000000000026518 34190185 PMC8257908

[32] Li F Gao Q Qiao E Yin G Zhang R Zhao S Contributing factor of proximal arch dilation in patients with bicuspid aortic valve-wall shear stress or upward extension of ascending aorta dilation? *Heart Surg Forum.* (2020) 23:E435–40. 10.1532/hsf.2925 32726222

[33] Mahadevia R Barker A Schnell S Entezari P Kansal P Fedak P Bicuspid aortic cusp fusion morphology alters aortic three-dimensional outflow patterns, wall shear stress, and expression of aortopathy. *Circulation.* (2014) 129:673–82. 10.1161/CIRCULATIONAHA.113.003026 24345403 PMC3946057

[34] Shan Y Li J Wang Y Wu B Barker A Markl M Aortic stenosis exacerbates flow aberrations related to the bicuspid aortic valve fusion pattern and the aortopathy phenotype. *Eur J Cardiothorac Surg.* (2019) 55:534–42. 10.1093/ejcts/ezy308 30215695 PMC6821178

[35] Stephens E Hope T Kari F Kvitting J Liang D Herfkens R Greater asymmetric wall shear stress in sievers’ type 1/LR compared with 0/LAT bicuspid aortic valves after valve-sparing aortic root replacement. *J Thorac Cardiovasc Surg.* (2015) 150:59–68. 10.1016/j.jtcvs.2015.04.020 25956338

[36] Bollache E Guzzardi D Sattari S Olsen K Di Martino E Malaisrie S Aortic valve-mediated wall shear stress is heterogeneous and predicts regional aortic elastic fiber thinning in bicuspid aortic valve-associated aortopathy. *J Thorac Cardiovasc Surg.* (2018) 156:2112–20.e2. 10.1016/j.jtcvs.2018.05.095 30060930 PMC6242768

[37] Guzzardi D Barker A van Ooij P Malaisrie S Puthumana J Belke D Valve-related hemodynamics mediate human bicuspid aortopathy: insights from wall shear stress mapping. *J Am Coll Cardiol.* (2015) 66:892–900. 10.1016/j.jacc.2015.06.1310 26293758 PMC4545965

[38] Li F Li X Wang Y Yu C Yin G Chen X The etiological heterogeneity of bicuspid aortopathy between ascending and root morphotype. *Heart Surg Forum.* (2020) 23:E913–9. 10.1532/hsf.3333 33399529

[39] Hope M Hope T Crook S Ordovas K Urbania T Alley M 4D flow CMR in assessment of valve-related ascending aortic disease. *JACC Cardiovasc Imaging.* (2011) 4:781–7. 10.1016/j.jcmg.2011.05.004 21757170

[40] Rodríguez-Palomares J Dux-Santoy L Guala A Kale R Maldonado G Teixidó-Turà G Aortic flow patterns and wall shear stress maps by 4D-flow cardiovascular magnetic resonance in the assessment of aortic dilatation in bicuspid aortic valve disease. *J Cardiovasc Magn Reson.* (2018) 20:28. 10.1186/s12968-018-0451-1 29695249 PMC5918697

[41] Meierhofer C Schneider E Lyko C Hutter A Martinoff S Markl M Wall shear stress and flow patterns in the ascending aorta in patients with bicuspid aortic valves differ significantly from tricuspid aortic valves: a prospective study. *Eur Heart J Cardiovasc Imaging.* (2013) 14:797–804. 10.1093/ehjci/jes273 23230276

[42] Piatti F Sturla F Bissell M Pirola S Lombardi M Nesteruk I 4D flow analysis of bav-related fluid-dynamic alterations: evidences of wall shear stress alterations in absence of clinically-relevant aortic anatomical remodeling. *Front Physiol.* (2017) 8:441. 10.3389/fphys.2017.00441 28694784 PMC5483483

[43] van Ooij P Garcia J Potters W Malaisrie S Collins J Carr J Age-related changes in aortic 3D blood flow velocities and wall shear stress: implications for the identification of altered hemodynamics in patients with aortic valve disease. *J Magn Reson Imaging.* (2016) 43:1239–49. 10.1002/jmri.25081 26477691 PMC4836971

[44] van Ooij P Potters W Collins J Carr M Carr J Malaisrie S Characterization of abnormal wall shear stress using 4D flow MRI in human bicuspid aortopathy. *Ann Biomed Eng.* (2015) 43:1385–97. 10.1007/s10439-014-1092-7 25118671 PMC4329118

[45] Callaghan F Grieve S. Normal patterns of thoracic aortic wall shear stress measured using four-dimensional flow MRI in a large population. *Am J Physiol Heart Circulatory Physiol.* (2018) 315:H1174–81. 10.1152/ajpheart.00017.2018 30028202

