# Supplementary figures and images for: 4D-flow MRI derived wall shear stress for the risk stratification of bicuspid aortic valve aortopathy: A systematic review
Source: Front Cardiovasc Med. 2023 Jan 9;9:1075833. doi: 10.3389/fcvm.2022.1075833 (PMC9869052; doi:10.3389/fcvm.2022.1075833)

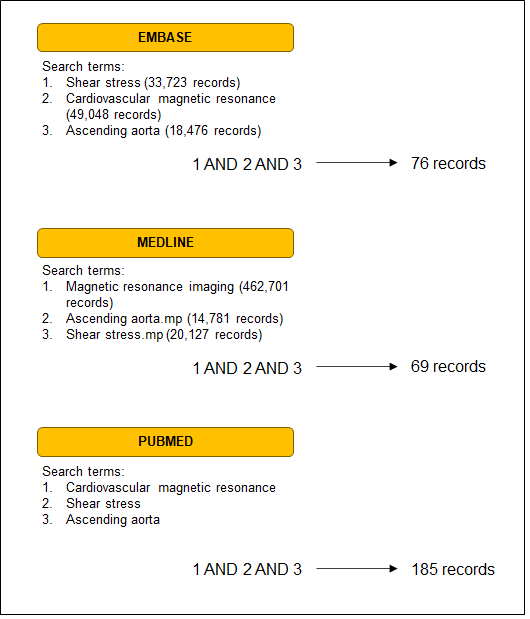

Supplement: Supplementary Figure 1 — Detailed database search strategy. [file Image_1.TIF]
