# Supplementary material for: 4D-flow MRI derived wall shear stress for the risk stratification of bicuspid aortic valve aortopathy: A systematic review
Source: Front Cardiovasc Med. 2023 Jan 9;9:1075833. doi: 10.3389/fcvm.2022.1075833 (PMC9869052; doi:10.3389/fcvm.2022.1075833)
Supplement: Supplementary file 2 [file Table_1.DOCX]

### **4D-flow MRI derived wall shear stress for the risk stratification of bicuspid aortic valve aortopathy: a systematic review**

**Authors:** Jiaxing Jason Qin^1,2^, Peyman Obeidy^1,2^, Mustafa Gök^1,2,3^, Alireza Gholipour^1,2^, Stuart M. Grieve^1,2*^

### **Table S1** Study list, population and key WSS objectives

| **Study** | **Study population/key WSS objectives** | **Comparator** | | |  | **Control** | | |  |
| --- | --- | --- | --- | --- | --- | --- | --- | --- | --- |
|  |  | **Age±stdv or range (years)** | **Total cases** | **Female cases** |  | **Age±stdv or range (years)** | **Total cases** | **Female cases** |  |
| Barker et al. 2012 [9] | WSS in BAV RL/RN vs controls (TAV - young health, age matched, age and aortic size matched) | 50±17 | 15 | 2 |  | Young: 23±2;  Age-matched: 67±8;  Age/size-matched: 53±19 | 15;  15;  15 | 3;  3;  2 |  |
| Bissell et al. 2013 [10] | WSS and flow pattern association with aortic dilatation in BAV vs healthy controls | 39.5±16.9 | 95 | 23 |  | 40.2±17.7 | 47 | 15 |  |
| Bollache et al. 2018 [36] | WSS and aortic wall histopathology correlation in BAV | 52±15 | 27 | 3 |  | NA | NA | NA |  |
| Dux-Santoy et al. 2020 [30] | WSS and role in aortic dilatation in BAV vs age-matched healthy controls | 45±14 | 46 | 19 |  | 43±13 | 44 | 19 |  |
| Farag et al. 2018 [21] | WSS association with AS and aortic diameter in BAV | 38.1±12.4 | 48 | 18 |  | 37.2±13.2 | 25 | 9 |  |
| Geeraert et al. 2021 [31] | WSS association with aortic dilatation in BAV vs healthy controls | 44±16 | 53 | 19 |  | 41±15 | 31 | 10 |  |
| Guala et al. 2022 [26] | Longitudinal evaluation of predictive value of WSS for aortic dilatation in BAV | 51±13 | 47 | 18 |  | NA | NA | NA |  |
| Guzzardi et al. 2015 [37] | WSS and aortic wall histopathology correlation in BAV | 48±15 | 20 | 2 |  | NA | NA | NA |  |
| Hope et al. 2011 [39] | WSS in BAV with normal flow vs BAV with helical flow vs healthy controls | Normal flow: 20.4±7.9;  Helical flow: 30.5±12.6 | 26 | 12 |  | 26.9±10.4 | 20 | 5 |  |
| Li et al. 2020a [38] | WSS association with elastic fibre thinning in BAV | 54.3±12.4 | 30 | 5 |  | NA | NA | NA |  |
| Li et al. 2020b [32] | WSS in BAV with dilated vs non-dilated ascending aorta | 57.9±7.8 | 16 | 5 |  | 50.7±14.4 | 18 | 2 |  |
| Mahadevia et al. 2014 [33] | WSS in BAV RL vs RN vs TAV with aortic dilatation vs healthy controls | RL: 44.7±8;  RN: 47.5±11.5 | 30 | 7 |  | 59.2±12.5 | 30 | 5 |  |
| Meierhofer et al. 2013 [41] | WSS in BAV vs healthy controls | 25 (10-44) | 18 | 8 |  | 25 (8-42) | 18 | 8 |  |
| Minderhoud et al. 2021 [27] | Longitudinal study of WSS and aortic growth with 3-year follow up in BAV | 34 (25-40) | 32 | 9 |  | 33 (28-48) | 28 | 13 |  |
| Piatti et al. 2017 [42] | WSS in BAV with normal valve function vs healthy controls | 25±10 | 5 | 1 |  | 23±7 | 10 | NA |  |
| Rahman et al. 2019 [28] | Longitudinal change in WSS in BAV with aortic dilatation vs TAV with aortic dilatation with >2-year follow up | 44.9±12 | 44 | 12 |  | 54.6±17 | 17 | 2 |  |
| Rizk et al. 2019 [25] | Diastolic WSS in BAV with AR vs healthy controls | 28 (19-34) | 19 | 1 |  | 26 (24-27) | 11 | 2 |  |
| Rodríguez-Palomares et al. 2018 [40] | WSS_cir_ and WSS_ax_ relationship with flow patterns in BAV | 47±13.2 | 101 | 37 |  | 50±16.4 | 20 | 5 |  |
| Shan et al. 2017 [22] | WSS in BAV with severe AR and AS vs BAV with mild AR and AS vs aorta size controlled TAV | AR: 46±7;  AS: 57±12 | 30 | 9 |  | 47±11 | 20 | 6 |  |
| Shan et al. 2019 [34] | Impact of AS on WSS in BAV cusp phenotypes and aortopathy phenotypes vs TAV | RL: 49±11;  RN: 50±10;  RL with AS: 59±7;  RN with AS: 56±8 | 120 | 44 |  | 54±10 | 20 | 7 |  |
| Soulat et al. 2022 [29] | Longitudinal study of surface area of elevated WSS association with aortic dilatation in BAV | 45±12 | 72 | 22 |  | 19-81 | 136 | 69 |  |
| Stephens et al. 2015 [35] | WSS in RL vs type0 in post valve sparing aortic root replacement vs TAV | 46.8±7.3 | 10 | 3 |  | 42.9±11.3 | 9 | 0 |  |
| van Ooij et al. 2016 [43] | Age impact on WSS comparing BAV vs age-matched healthy controls | Younger: 25±3;  Older: 54±2 | 20 | 7 |  | 44±13 | 56 | 19 |  |
| van Ooij et al. 2017 [23] | WSS in BAV with AS vs TAV with aortic dilatation and AS | 37-59 | 280 | 74 |  | 61 (52-70) | 245 | 49 |  |
| van Ooij et al. 2015 [44] | WSS heatmap in BAV compared to population average of healthy controls | 51±17 | 13 | 0 |  | 50±14 | 10 | 4 |  |
| von Knobelsdorff-Brenkenhoff et al. 2016 [24] | WSS in BAV and TAV with AS vs healthy control | 63±13 | 37 | 14 |  | 60±10 | 37 | 17 |  |

AR: aortic regurgitation; AS: aortic stenosis; BAV: bicuspid aortic valve; RL: BAV RL phenotype; RN: BAV RN phenotype; TAV: tricuspid aortic valve; WSS: wall shear stress; WSS_ax_: axial WSS; WSS_cir_: circumferential WSS.
